# Ongoing controversies surrounding cardiac remodeling: is it black and white—or rather fifty shades of gray?

**DOI:** 10.3389/fphys.2015.00202

**Published:** 2015-07-22

**Authors:** Sebastian Spaich, Hugo A. Katus, Johannes Backs

**Affiliations:** ^1^Research Unit Cardiac Epigenetics, Department of Cardiology, Angiology and Pneumology, University of HeidelbergHeidelberg, Germany; ^2^German Centre for Cardiovascular Research, Partner Site Heidelberg/MannheimHeidelberg, Germany; ^3^Department of Cardiology, Angiology and Pneumology, University of HeidelbergHeidelberg, Germany

**Keywords:** cardiac remodeling, plasticity, calcineurin, PKA, CaMKII, signaling, protein turnover

## Abstract

Cardiac remodeling describes the heart's multimodal response to a myriad of external or intrinsic stimuli and stressors most of which are probably only incompletely elucidated to date. Over many years the signaling molecules involved in these remodeling processes have been dichotomized according to a classic antagonistic view of black and white, i.e., attributed either a solely maladaptive or entirely beneficial character. By dissecting controversies, recent developments and shifts in perspective surrounding the three major cardiac signaling molecules calcineurin (Cn), protein kinase A (PKA) and calcium/calmodulin-dependent kinase II (CaMKII), this review challenges this dualistic view and advocates the nature and dignity of each of these key mediators of cardiac remodeling as a multilayered, highly context-sensitive and sophisticated continuum that can be markedly swayed and influenced by a multitude of environmental factors and crosstalk mechanisms. Furthermore this review delineates the importance and essential contributions of degradation and proteolysis to cardiac plasticity and homeostasis and finally aims to integrate the various aspects of protein synthesis and turnover into a comprehensive picture.

## Introduction

Heart failure is the leading cause of death in industrialized countries (Nichols et al., 2014). It is generally preceded by remodeling processes to adapt to alterations in wall tension in the myocardium or stress by external (hormonal) stimuli. In this regard, cardiac remodeling describes the heart's fascinating capability to respond and adapt to various stimuli. The mechanisms of cardiac plasticity, i.e., the potential of the heart to shrink or grow, are tremendous as the dynamic growth range of the myocardium exceeds 100% (Hill and Olson, 2008). Successful adaption to and adequate reduction of increased wall tension are ultimate goals of cardiac remodeling and facilitating preservation or even augmentation of cardiac pump function (Frey and Olson, 2003; Hill and Olson, 2008). The multifaceted mechanisms of cardiac remodeling and plasticity have traditionally been divided into beneficial (or “physiological”) or maladaptive (or “pathological”) hypertrophic remodeling of the heart on the one hand and cardiac atrophy on the other hand.

While cardiac growth during maturation (“postnatal hypertrophy”), maternal cardiac growth during pregnancy and exercise-induced cardiac hypertrophy are all considered physiological entities of hypertrophy, pathological hypertrophic responses are observed upon sustained neurohumoral activation and abnormal mechanical stretch of the myocardium as common findings of various cardiac disease entities. These include but are not limited to ischemic events (myocardial infarction), pressure overload conditions such as arterial hypertension and aortic valve stenosis, genetic disorders due to alterations in key sarcomeric or metabolic proteins as well as infectious and toxic triggers (Hill and Olson, 2008; Kehat and Molkentin, 2010; van Berlo et al., 2013; Tham et al., 2015). Atrophic remodeling is generally observed in patients with protracted bed rest and ventricular unloading as well as malignant disease (Hill and Olson, 2008; Cosper and Leinwand, 2011; Springer et al., 2014).

Physiological hypertrophy of the heart—as it is observed in postnatal growth as well as in response to pregnancy and exercise—is characterized by a fine-tuned and orchestrated process of beneficial adaptations. These modulations result in decreased cardiac wall stress, augmentation of pumping performance and improvements of vascularization, while maladaptive effects of the increased workload that lead to hypertrophic remodeling are countered and kept at bay. Various signaling molecules and pathways have been shown to take part in these adaptive processes and several excellent reviews have summarized our present knowledge on this physiological aspect of cardiac remodeling (Hill and Olson, 2008; Maillet et al., 2013). Generally considered as beneficial, these pathways are triggered by endocrine mediators or mechanosensors governing a number of adaptive intracellular signaling cascades. Ligands that result in physiological downstream signaling include vascular endothelial growth factor B (VEGF-B), insulin, growth hormone (GH) and insulin-like growth factor (IGF1) as well as the thyroid hormone triiodothyronine (T3) (Yoshida et al., 2010; Maillet et al., 2013; Tham et al., 2015). Via their respective receptors, these stimuli converge on downstream pathways controlling the induction of adaptive gene programs and protein synthesis, and direct cellular metabolism and energy production. Well-characterized cascades and pathways regulating these homeostatic mechanisms comprise phosphoinositide 3 kinase (PI3K)/AKT, mTOR complex 1 (mTORC1), ERK1/2 and AMP-activated protein kinase (AMPK) (Baker et al., 1993; Liu et al., 1993; McMullen et al., 2003; Seth et al., 2009; Bostrom et al., 2010; Zhang et al., 2010). Translating stretch-stimuli to downstream signaling, the mechanosensing apparatus is controlled by transient receptor potential (TRP) channels, integrins and various z-disc associated proteins such as muscle LIM protein (MLP), actinin-associated LIM protein (ALP) and Nebulette (NEB) as well as the sarcomere-spanning protein titin (Linke, 2008; Seth et al., 2009; Frank and Frey, 2011; Luedde et al., 2011; Hamdani et al., 2013; Maillet et al., 2013). While the notion of (these) mediators as the “good guys” in cardiac remodeling seems very appealing and is founded on robust scientific work(s), a note of caution seems advisable not to fall for a false dichotomy bearing in mind what Paracelsus postulated about 500 years ago: “Dosis sola venenum facit” (Shiojima et al., 2005; Hill and Olson, 2008; Tham et al., 2015).

Pathological hypertrophic remodeling on the other hand—while initially compensatory by reduction of ventricular wall stress and temporal preservation of cardiac pump function—eventually evolves into a devastating spiral of maladaptive alterations culminating in heart failure and death (Hill and Olson, 2008; Kehat and Molkentin, 2010; Burchfield et al., 2013; Molkentin, 2013; Tham et al., 2015). In this regard, pathological hypertrophic remodeling is characterized by multiple facets of ultimately maladaptive mechanisms such as altered calcium handling, changes in metabolic patterns and gene expression, affection of individual cell fate and survival as well as modifications of the extracellular environment by fibrosis. Cardiac stress by increased neurohumoral activation (mainly endothelin 1, angiotensin II and catecholamines) and abnormal mechanical stretch converge on a number of sensors (mostly G-proteins and strain-sensitive cellular elements) which translate these stimuli into respective stress response pathways (Hill and Olson, 2008; Kehat and Molkentin, 2010; Burchfield et al., 2013; Molkentin, 2013; Tham et al., 2015). Key second messengers that were suggested in this regard include calcium, phospholipase C, guanylyl, and adenylyl cyclases. Downstream kinases, phosphatases and other enzymes with critical roles in pathologic remodeling comprise calcineurin, protein kinase A (PKA) and C (PKC), cGMP-dependent protein kinases (PKG) calcium-calmodulin-dependent kinase II (CaMKII) and mitogen-activated protein kinases (MAPKs) with their respective downstream components—to name but a few (Benovic et al., 1986; Molkentin et al., 1998; Allen and Leinwand, 2002; Backs et al., 2006, 2009; Casey et al., 2010; Kehat and Molkentin, 2010; Burchfield et al., 2013; van Berlo et al., 2013; Kreusser et al., 2014; Tham et al., 2015).

Several outstanding reviews have been written in recent years dissecting details of these mediators and pathways integral to maladaptive/pathological remodeling processes (Kehat and Molkentin, 2010; Burchfield et al., 2013; van Berlo et al., 2013; Bisping et al., 2014; Tham et al., 2015). Therefore this review will focus on a very limited number of crucial effectors highlighting new aspects and shifts in perspective that might challenge long-standing dogmas.

Notably, RNA-based signaling pathways and mediators as well as epigenetic and other posttranslational modifiers are currently evolving as further crucial contributors to cardiac remodeling. However, elaborations on these developments would transcend the scope of this review. Furthermore they have extensively been covered in recent publications and reviews that focused on each of the above issues individually (Papait et al., 2013; Lehmann et al., 2014; Liu et al., 2015; Gillette and Hill, 2015; Philippen et al., 2015; Piccoli et al., 2015; Thum and Condorelli, 2015; Uchida and Dimmeler, 2015).

## Allegory: cardiac remodeling as tuning of a machine

Allegorically, cardiac plasticity could be referred to as the maintenance and adjustment of a machine subjected to heavy and undulating workload. The machine (*heart*) can be optimized (*remodeled*) by technicians (*key mediators*) to perform according to the present demand by employing different fuel (*metabolic*), spare parts and logistics (*structural and functional alterations*). These remodeling efforts can either be construed as long-lasting, sustainable and deliberate optimizations (as it is generally observed in physiological hypertrophy), or endeavors can be targeted at drastic, short-term improvements which will not be sustainable for a longer period of time but provide immediate and powerful responses to increased demands (as observed in many aspects of pathological hypertrophy). Furthermore, it appears crucial whether the demand will have to be fulfilled infinitely (analogous to many pathological stress models) or whether periods exist when the machine can (temporarily) cool down and reduce its output (as in exercise-related hypertrophy).

Atrophy on the other hand could be seen as dismantling of a machine and its appendices in response to reduced demand. This dismantling process can be achieved by a multitude of different ways depending on focus and technique of involved technicians and parties.

Finalizing this allegorical approach, the technicians (*key mediators and signaling molecules*) driving these changes of the machine may be similar or even the same in various aspects of remodeling. However, their intentions, perspectives and approaches may be different depending on a vast number of environmental, extrinsic and intrinsic factors.

Translating this simile to cardiac pathophysiology, it is our opinion that key protagonists in cardiac plasticity may not as easily be characterized as “good” or “bad,” beneficial or maladaptive. To our minds, these essential mediators may exert different functions and adaptations depending on timing, duration and dosage of respective stimuli as well as crosstalk with other crucial protagonists that are simultaneously activated leading to numerous distinct posttranslational modifications.

**Figure 1:**
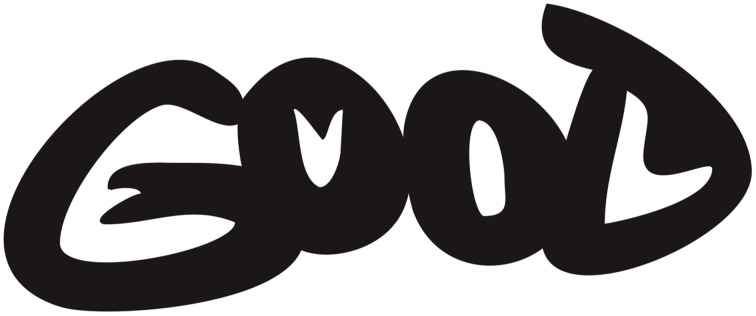
**Illustration of ***good*** and ***evil*** as an optical illusion/ambigram**. This illustration of the words *good* and *evil* in the context of an optical illusion is implemented to emphasize two things: 1. Expectations drive our perception and may therefore create a bias, i.e., if we are looking for something positive, we will probably recognize the word *good* first. If negative expectations prevail, the word *evil* will most likely be seen first. In this regard, we would like to emphasize that research efforts are prone to hold to the same pattern and this bias has to be mindfully dealt with and kept in mind. 2. The idiomatic phrase of “two sides to every coin” is reflected in the concomitant depiction of *good* and *evil* serving as a simile that key mediators of cardiac remodeling processes cannot be dichotomized in solely good or bad protagonists as the nature and dignity of their signaling will range a context-sensitive continuum with positive *(good)* and negative *(evil)* results. *Good-Evil Ambigram designed by and courtesy of Punya Mishra (punyamishra.com)*.

For instance, the phosphatase calcineurin and its role in cardiac remodeling have been studied for almost two decades now (Molkentin et al., 1998; Molkentin, 2013). While it was initially described and for a long time considered only as key mediator of pathological hypertrophy and remodeling, evidence has been accumulating in recent years that calcineurin might also play an important role in physiological cardiac growth (Heineke et al., 2010a; Felkin et al., 2011; Chung et al., 2013; Hudson and Price, 2013; Molkentin, 2013; Kreusser et al., 2014; Mattiazzi and Kranias, 2014). The resulting scientific discussion whether calcineurin is the “bad guy” in cardiac growth nicely illustrates how complex cardiac remodeling actually is: it raises the question, whether the sophisticated means of cardiac hypertrophy and remodeling can simply be broken down into bad or good, black or white, or whether we should not consider the multifaceted and finely tuned cardiac response mechanisms as a continuum (of shades of gray) (Figure 1).

To our minds, future research should therefore concentrate on elucidating the myriad of crosstalk mechanisms between the currently identified main pathways and key protagonists. This will enable us to meticulously modulate the major signaling pathways and molecules by augmenting those sidetracks that are desirable in response to a given stressor, while repressing those that perturb or prohibit adequate cardiac homeostasis.

While there is broad consensus on the fact that hypertrophy and atrophy can be either beneficial or maladaptive, the exact triggers, mediators and pathways leading to these distinct entities are under constant debate. In this regard, several mediators that had been classified as maladaptive have been rehabilitated over the years, whereas seemingly benign players have been attributed potential “dark sides” (Heineke et al., 2010a; Peng et al., 2010; Felkin et al., 2011; Chung et al., 2013; Zhang et al., 2013; Kreusser et al., 2014; Li et al., 2014; Mattiazzi and Kranias, 2014; Tham et al., 2015).

As cardiac remodeling mechanisms involve a myriad of different protagonists and interactions, this review will focus on ongoing controversies and current developments surrounding adaptive vs. maladaptive signaling in cardiac remodeling (Calcineurin, PKA, CaMKII) as well as shifts in perspective in terms of relative importance of select mechanisms in cardiac remodeling (namely proteasomal/degradation mechanisms).

## Calcineurin-signaling

Calcineurin (Cn), one of the heart's most intensively studied enzymes, is a calcium-dependent serine/threonine phosphatase controlling key functions and processes in response to a wide range of stress stimuli. Calcineurin is composed as a heterodimer of catalytic (CnA) and smaller regulatory subunits (CnB) with CnA-alpha and CnA-beta as well as CnB1 constituting the relevant myocardial isoforms (Taigen et al., 2000; Bueno et al., 2002). Generally activated by calcium (or to a lesser extent calpain-cleavage), calcineurin dephosphorylates nuclear factor of activated T cells (NFAT) as its major downstream target (Molkentin et al., 1998; Hogan et al., 2003; Wilkins et al., 2004). Upon dephosphorylation, NFAT in turn translocates to the nucleus where it orchestrates the re-activation of fetal genes such as atrial natriuretic factor (ANF), brain natriuretic peptide (BNP) and skeletal actin (SA) as part of the prototypical cardiac stress response (Molkentin et al., 1998; Hogan et al., 2003; Frey et al., 2004; Wilkins et al., 2004; Frank et al., 2007). While it is important to note that this activation of the fetal gene program principally constitutes adaptive efforts to cope with the increased cardiac stress, it is still generally perceived as an epiphenomenon and utilized as a marker of unresolved and (therefore in the long run) pathological stress.

Almost two decades ago, Molkentin and Olson—in a very elegant study—first described calcineurin as a key mediator of pathologic cardiac hypertrophy and consecutive progression to heart failure (Molkentin et al., 1998). In the following, numerous studies elaborated on the role of calcineurin in cardiac hypertrophy and remodeling as well as heart failure. These well conducted studies provided overwhelming evidence that calcineurin is indeed at the center of cardiac hypertrophy and remodeling (Zou et al., 2001; Hill et al., 2002; Wilkins et al., 2004; Molkentin, 2013; van Berlo et al., 2013). In this regard, loss of function models by either targeting calcineurin directly (Zou et al., 2001; Bueno et al., 2002) or one of its modulators such as regulator of calcineurin (RCAN), AKAP-79 or Cabin-1 (De Windt et al., 2001; Rothermel et al., 2001; Hill et al., 2002) established beyond doubt that downregulation of calcineurin prevented cardiac hypertrophy.

Moreover, data from most experiments on pharmacological inhibition of calcineurin fortified this notion (Sussman et al., 1998; Burchfield et al., 2013; Molkentin, 2013). Consequently, overexpression studies with either activated calcineurin (Molkentin et al., 1998) or knockdown of its negative modulators (Frey et al., 2004) provided the complimentary picture of increased susceptibility to heart failure in the wake of exaggerated (pathological) hypertrophy (Molkentin, 2013).

Therefore, the vast majority of experiments led to the general opinion that calcineurin is the “bad guy” in cardiac remodeling, following the classical black-and-white dualism of attributing either solely “pathological” or “physiological” qualities and functions to a given cellular component or signaling pathway. In recent years, this (false) dichotomy has evolved into a more sophisticated view of calcineurin in particular and cardiac remodeling in general (Heineke et al., 2010a; Felkin et al., 2011; Heineke and Ritter, 2012; Chung et al., 2013; Molkentin, 2013; Seto et al., 2013; Kreusser et al., 2014). In this regard, the entirely black (-and-white) picture has evolved as several shades of gray have been elucidated in calcineurin's role.

Although robust scientific evidence demonstrates the maladaptive nature of many pathways on which calcineurin has a major impact, a number of studies on protective aspects of calcineurin-mediated signaling have accumulated over the past decade. Furthermore, one pitfall from studies on regulators of calcineurin, that deserves mentioning, concerns the notion that these regulators of calcineurin may not only or primarily target calcineurin, but may very well also affect other important signaling mediators.

While first evidence of protective aspects of calcineurin signaling was already observed in terms of anti-apoptotic properties by De Windt et al. (2000), a number of studies in the years to follow fortified the notion that calcineurin may indeed provide beneficial signaling under special circumstances and should not be reduced to the maladaptive and destructive culprit (Bueno et al., 2004; Frey et al., 2008; Heineke et al., 2010a; Chung et al., 2013; Kreusser et al., 2014). These studies rather served as a warning against demonizing calcineurin in the wake of compelling data on maladaptive aspects of excessive calcineurin activity.

Consistent with this notion of a context-sensitive dignity of calcineurin signaling, a knock-out study of calsarcin-2, a negative modifier of calcineurin activity in skeletal muscle, even demonstrated enhanced exercise performance in absence of a myopathic phenotype (Frey et al., 2008), while deletion of calsarcin-1, the cardiac-enriched isoform repressing calcineurin activity, resulted in accelerated cardiomyopathy and heart failure due to exaggerated calcineurin signaling (Frey et al., 2000, 2004, 2008). These exciting observations hinted at multiple layers of calcineurin signaling with physiological facets.

Intriguingly, Leinwand and colleagues published data on the importance of calcineurin in regard to cardiac hypertrophy during pregnancy, another classical prototype of physiological cardiac remodeling (Chung et al., 2013). In particular, they show calcineurin to be upregulated in pregnant mice as part of physiological cardiac remodeling during early pregnancy. Their observation of biphasic regulation of calcineurin during pregnancy supports the idea that timing and intensity of a stimulus and its responsive signaling cascades might be more important than the individual effectors themselves in terms of long-term consequences of the respective stimuli (Chung et al., 2013). Furthermore, their observations strengthen the notion that no single mediator, but crosstalk between signaling cascades (specifically calcineurin, ERK and Akt as well as hormonal balances) constitutes the paramount principle governing cardiac response mechanisms to the physiologic remodeling stimuli induced by pregnancy. Therefore, context and duration of calcineurin activation may be the key determinants whether calcineurin exerts its predominantly negative effects on cardiac remodeling pathways or whether it contributes to adaptive and beneficial signaling cascades in response to stressors (Chung et al., 2013).

Insight on regulation of the nature of calcineurin signaling has also been provided by Kreußer et al., illustrating calcineurin as a key contributor to various entities of hypertrophy, but CaMKII as a major mediator in conducting maladaptive stimuli (Kreusser et al., 2014). Indeed, calcineurin appears to be (co-)regulated by CaMKII. While co-activation during pathological stress results in significant impairment of cardiac function in the wake of cardiac remodeling, deletion of the two cardiac CaMKII genes δ and γ in this setting is sufficient to generate a cardiac phenotype that resembles a more physiological nature (calcineurin-dependent cardiac hypertrophy without significant systolic dysfunction). This observation provides evidence for the conclusion that calcineurin—while contributing to hypertrophic remodeling—may confer beneficial changes in a context-dependent response to external stressors (Kreusser et al., 2014).

Observations from studies by Lara-Pezzi's group add another layer of complexity, as they show induction of a splicing variant of calcineurin (CnAβ1) to be protective after myocardial infarction (Felkin et al., 2011). These findings hint at intricate and sophisticated switching mechanisms in response to cellular stressors emphasizing that no single pathway, but the interactions of multiple cascades may determine remodeling to be adaptive or maladaptive.

In an interesting review, Heineke and Ritter elaborate on the concept of compartment/subdomain-specific modification of calcineurin activity. This would allow for context-dependent physiological or pathological means of remodeling (Heineke and Ritter, 2012). Work on the calcium and integrin-binding-protein-1 (CIB1) exemplarily illustrate how differential binding and translocation of important cellular signaling mediators (in this case calcineurin) affect the nature of downstream effects and course of remodeling by compartmentalization (Heineke et al., 2010). It is still unclear to what degree this concept of “fishing for calcium” by different mediators of stress signals can unravel the mysteries of the diverse characters of cardiac remodeling.

Looking further downstream at possible modifiers of calcineurin signaling that may sway the dignity of a stimulus, interactions with other transcription factors (such as MEF2, AP-1, GATA4) as effectors of multiple upstream cascades modify NFAT-dependent DNA-interaction and integrate various pathways into meticulously tuned stress responses (Hogan et al., 2003; Heineke et al., 2010; Heineke and Ritter, 2012).

Taken together, it is well established that calcineurin is at the center of cardiac hypertrophy. However, evidence is accumulating that calcineurin's role may very well be part of a continuum of (fifty or more) shades of gray in cardiac remodeling rather than confined to the black-and-white dualism that dominated the last decades of research in the field of cardiac remodeling.

## Protein kinase a—signaling in cardiac remodeling

Protein kinase A or cyclic AMP-dependent Kinase (PKA) constitutes the canonical downstream effector of the β-adrenergic cascade in myocardial signaling. Activation of the beta-adrenergic receptor (βAR), perhaps the heart's most crucial subclass of G-protein coupled receptor (GPCR), by catecholamines primarily elicits an increase in cellular cAMP via induction of cytosolic adenylyl cyclase (Benovic et al., 1986; Xie et al., 2013; van Berlo et al., 2013; Tham et al., 2015). Consequently, a number of critical signaling chains and downstream effectors are activated, contributing greatly to the immediate cardiac stress response (“fight or flight” concept) by boosting myocyte inotropy, chronotropy, and lusitropy. Mechanistically, elevation of cAMP predominantly results in activation of PKA. Activated PKA in turn rules over numerous critical downstream pathways by phosphorylating pivotal calcium-handling proteins and crucial parts of the myocytes' contractile apparatus targeted at optimization of cardiac contractility and (augmented) function. In this regard, PKA is at the heart of the myocytes' signaling pathways which integrate stimuli and stressors into orchestrated cascades that enhance cardiac remodeling and therefore ensure short- and long-term homeostasis and integrity of cellular functions (Benovic et al., 1986; Backs et al., 2011; Burchfield et al., 2013; Xie et al., 2013; van Berlo et al., 2013; Tham et al., 2015). As tremendous efforts in PKA research have elucidated and meticulously characterized a myriad of aspects of PKA signaling, this review can only highlight some recent frontiers, controversies and shifts in perspective. It aims to pick up on the notion that—with increasing knowledge—simplified models might have to make room for concepts of multi-layered and meticulously tuned processes, since key players themselves and associated cellular pathways prove even more complex than initially perceived.

Therefore, this review will focus on the following aspects of cardiac remodeling as far as PKA is concerned: importance of duration and alternate means of PKA stimulation/activation, and (in the following section) crosstalk of PKA with CaMKII, perhaps the most intricately intertwined protagonists and pathways of myocyte remodeling.

The (erratic) viewpoint of PKA as a detrimental player in cardiac remodeling and heart failure was significantly stimulated by the adverse cardiac phenotype observed in a transgenic mouse model overexpressing constitutively active PKA (Antos et al., 2001). In the years to follow, a more sophisticated picture of the role of PKA in the myocyte's stress response and remodeling processes emerged as the significance of dosage, time dependency and hierarchic crosstalk with other mediators such as CaMKII became apparent. In this regard, it became clear that PKA elicits protective effects upon immediate β-adrenergic stimulation, e.g., by phosphorylation of sarcomeric structures such as titin, while transgression to heart failure was actually found to be associated with decreased phosphorylation of PKA dependent targets. These findings fortified the idea that PKA might have a more pronounced role in early remodeling as opposed to chronic stress (Yamasaki et al., 2002; Kruger and Linke, 2006; Grimm and Brown, 2010; Backs et al., 2011; Fischer et al., 2013a).

In line with these observations, short-term stimulation of the β-adrenergic cascade confers a number of protective effects and mediates essential “fight or flight” responses. Chronic stimuli cause the remodeling processes to turn into a detrimental spiral of beta-receptor desensitization and pathologic hypertrophy with subsequent development of heart failure. Long-term β-adrenergic stimulation leads to negative modulation of the respective receptors as well as downstream effectors and targets by a number of key regulators, but the exact mechanisms of this duration-dependent switch in effects of beta-adrenergic stimulation and stress are incompletely understood (Engelhardt et al., 1999; Bristow, 2000; Hill and Olson, 2008; Zhang et al., 2013; van Berlo et al., 2013). However, it is widely accepted that GPCR receptor kinases (GRKs) constitute a pivotal class of enzymes which restrict the dynamic range of βAR-function by phosphorylation of the receptors themselves and by modification of beta-arrestin signaling, a side-track of the β-cascade which is crucial for protective downstream effects (Strulovici et al., 1984; Benovic et al., 1986). Targeting of these duration-dependent negative regulators offers a promising approach to take advantage of PKA-mediated increases in cardiac contractility while repressing those intermediates and modulators in the PKA-cascade that lead to detrimental remodeling and subsequent heart failure (van Berlo et al., 2013). For instance, blocking GRK2 interaction with the GPCR alleviates pathologic remodeling, whereas amplified contractility is still observed (Casey et al., 2010).

The second aspect of PKA that we would like to zero in on in this review is the means by which PKA gets activated, as this seems to play a critical role in downstream effects. Furthermore, it illustrates that various environmental stimuli may divergently modify the character and dignity of remodeling and therefore induce distinctly different phenotypes, although they employ one and the same kinase. The above depicted β-adrenergic cascade serves as the predominant extracellular means to activate PKA. However, a number of alternate mechanisms have been proposed with reactive oxygen species (ROS) as the most intriguing elicitor (Brennan et al., 2006; Sag et al., 2013; van Berlo et al., 2013; Wagner et al., 2014; Tham et al., 2015).

Reactive oxygen species (ROS) and respective sensors and counterparts are integral parts of (sub)cellular signaling in many compartments of each cell. They contribute greatly to the myocyte's stress response and remodeling efforts (Burgoyne et al., 2013; Sag et al., 2013; Wagner et al., 2014; Tham et al., 2015). In this regard, ROS can either directly affect target structures by oxidation or trigger a number of enzymes to modify downstream effectors leading to altered calcium signaling and contractility, gating of ion channels as well as differential regulation of homeostatic mechanisms concerned with trophy, metabolism and cell survival (Burgoyne et al., 2013; Sag et al., 2013; Wagner et al., 2014; Tham et al., 2015).

Therefore, amongst others a publication from Phil Eaton's group deserves special attention. In multiple well-conducted experiments they demonstrate dose-dependent activation of PKA by dimerization of its regulatory subunit and consecutive compartmentalized modification of respective PKA targets. Notably, this activation of PKA by H2O2 was independent of cAMP levels, the mediator of βAR-induced PKA activation (Brennan et al., 2006). Interestingly, the study provides evidence that the postulated targets of oxidant-induced PKA are at least in part congruent with those phosphorylated upon classic beta-adrenergic activation of PKA. While many of the mechanistic details of this alternate pathway of PKA activation remain elusive, it has become evident that oxidant-induced activation confers a means of targeting PKA to its subcellular compartments and inducing its kinase activity in these select environments. Furthermore, these findings imply that the effects of PKA may be dependent on and vary with multiple environmental factors (β-adrenergic stimulation, ROS, intracellular calcium, etc.) whose crosstalk and relative contributions may determine PKA's quintessential role in any given situation. For instance, ROS-induced activation of PKA—similarly to beta-adrenoreceptor-mediated induction of PKA—results in an increase of cardiac contractility. However, the degree, dosage and duration of ROS stress appear to strongly influence these remodeling mechanisms as strong and long-lasting ROS stimuli seem to reverse the aforementioned pro-inotropic effects (Goldhaber and Liu, 1994; Yao et al., 2003). Suggesting another regulatory principle compatible to the previously described differential effects of different isoforms in calcineurin signaling, Brennan and colleagues also speculate on the possibility that redox-sensitivity applies mainly for type I PKA, while the type II entity may be more prone to β-adrenergic signaling (Brennan et al., 2006; Felkin et al., 2011).

Therefore this example of differential activation of PKA by ROS (as well as the regulatory mechanisms in terms of stability and degradation touched upon later in this review) may serve as another illustration of the complexity of cardiomyocyte signaling and remodeling mechanisms.

## CaMKII-signaling in cardiac remodeling

Calcium-Calmodulin-dependent Kinase II (CaMKII) is at the center of multiple cardiac signaling cascades and modulates the heart's response to various extrinsic and intrinsic stimuli (Anderson et al., 2011; van Berlo et al., 2013; Grandi et al., 2014; Kreusser et al., 2014; Kreusser and Backs, 2014; Mattiazzi and Kranias, 2014; Weinreuter et al., 2014; Tham et al., 2015). The main cardiac isoforms of CaMKII, gamma and delta, have been studied in depth in terms of their role in cardiac remodeling processes. As several excellent reviews (Anderson et al., 2011; Maier, 2012; Fischer et al., 2013b; Herren et al., 2013) and a very comprehensive topic series on CaMKII (synopsis in Grandi et al., 2014) have extensively covered the spectrum of CaMKII in cardiac physiology and pathology only very recently, the scope of this review will limit its attention to interactions and crosstalk with the aforementioned protagonists of cardiac signaling, calcineurin and PKA, as well as emerging twists and desiderata of CaMKII research.

In regard to the latter, CaMKII activation requires calcium and calmodulin to dissolve the autoinhibitory conformation of CaMKII, thereby exposing both the regulatory and catalytic domain of the molecule. In the wake of prolonged activation, autophosphorylation at T287 occurs as a major posttranslational mechanism to stabilize CaMKII in its activated form (Meyer et al., 1992; Erickson, 2014). In recent years, a number of studies have uncovered multiple alternate posttranslational modifications of CaMKII (especially of the regulatory domain and mainly activating in nature) offering a new conundrum as to the contributions and interdependency of these various regulatory mechanisms (Erickson, 2014). Select principles of this divergent activation of CaMK include oxidation, glycosylation and nitrosylation as well as interactions with intracellular proteins (such as alpha-actinin or the NMDA receptor in the brain) (Jalan-Sakrikar et al., 2012; Burgoyne et al., 2013; Erickson et al., 2013; Sag et al., 2013; Coultrap and Bayer, 2014; Erickson, 2014).

As with calcium, the previously mentioned activation of CaMKII via ROS seems especially intriguing as it parallels the induction of PKA by ROS. This parallelism construes another enigma in terms of the hierarchical, spatial and temporal patterns and regulatory mechanisms that determine which of the two kinases is preferentially targeted and therefore supreme in its effects at any given time. Furthermore, this ROS/oxidant-dependent co-induction of CaMKII and PKA is especially enthralling, as both kinases do not only share a great number of binding partners and downstream effectors such as sarcomeric proteins (e.g., cMyBPC, actinin), calcium handling proteins (RyR, PLN) as well as various calcium and sodium channels (e.g., NaV1.5), but sometimes even phosphorylate a protein at the exact same amino acid position (Faul et al., 2007; Anderson et al., 2011; Herren et al., 2013; Sag et al., 2013; Dobrev and Wehrens, 2014; Mattiazzi and Kranias, 2014; Wagner et al., 2014).

In some instances, this mystery of multiplicity, redundancy or even competition of PKA and CaMKII has been partially unraveled, as PKA was demonstrated to regulate histone deacetylase 4 (HDAC4) by inducing proteolysis, thereby selectively repressing myocyte enhancer factor-2 (MEF2) activity and shutting out CaMKII signaling tracks (Backs et al., 2011). Interestingly, this modulation of HDAC4 confers a switch toward a more protective cascade by favoring SRF over MEF2 as HDAC4-NT's primary target structure. In this way it serves as a gateway disrupting sidetracks that would cause the nature of cardiac remodeling to take a maladaptive direction (Backs et al., 2011).

A second example of progress in this area refers to the relative importance of the key signaling mediators calcineurin and CaMKII in regard to their roles in (maladaptive) cardiac remodeling. As previously delineated both signaling pathways (CaMKII and calcineurin) critically contribute to the pressure overload-induced phenotype of adverse remodeling, but knockout of both cardiac CaMKII genes demonstrates the supremacy of CaMKII for the maladaptive nature of remodeling in this setting, while calcineurin—when isolated from CaMKII crosstalk and interdependency—also seems to trigger beneficial processes in terms of a more physiological remodeling phenotype (Kreusser et al., 2014).

However, passing the buck to CaMKII and thereby advocating it as the black sheep in terms of cardiac remodeling does not seem justified since experimental evidence points at more intricate and multi-layered regulatory mechanisms. For instance, the isoform or subtype dependent variances in downstream signaling described for CaN and PKA have also been proposed for CaMKII and may significantly complicate and impede simplified explanations following a dualistic projection (Peng et al., 2010; Mishra et al., 2011; Gray and Heller Brown, 2014).

To further elaborate on the enigma of multiplicity, redundancy or even competition, the multi-adaptor z-disc protein myopodin has not only been shown to be phosphorylated by both PKA and CaMKII (both resulting in nuclear import of myopodin), but has been shown to be dephosphorylated by calcineurin, the third pivotal mediator of cardiac remodeling covered in this review (Faul et al., 2007; Linnemann et al., 2010). Similar data in terms of unresolved redundancy and/or multiplicity exists in regard to calcium or sodium handling proteins such as the RyR or the NaV1.5 channel (Camors and Valdivia, 2014; Dobrev and Wehrens, 2014; Grandi and Herren, 2014). While most of the biological implications and functions of these tantalizing observations remain elusive so far, these findings are nevertheless illustrative of the concept that key players in cardiomyocyte signaling significantly intertwine with their intermediates and downstream effectors so that dichotomizing protagonists in dualistic cascades may not always be as helpful as initially thought.

Deciphering crosstalk of CaMKII and PKA as two of the major signaling kinases in the heart has intrigued scientists for years and led to elucidation of various intermediates which constitute indirect signaling loops and pathways that integrate signals from one of these cascades into the other and modulate the response of one kinase depending on the other's signaling.

Exemplarily, recent works from Mika and colleagues demonstrate a feedback loop in which CaMKII phosphorylates PDE4D, which in turn leads to downregulation of cytosolic cAMP levels and consequent inactivation of PKA (Mika et al., 2015). Interestingly, although both kinases show this variety of identical interaction partners and targets, direct interaction and modulation of one of the kinases by the other has never been demonstrated to date.

In summary, there is broad consensus in the scientific community that factors like duration, intensity and environmental setting of extrinsic and intrinsic stimuli are most crucial for the downstream mediation of cardiac remodeling. In this regard, the realization of signaling pathways and single effectors integrating into a myriad of complex interactions and crosstalk-dependent downstream effects have led to the belief that these mechanisms of fine-tuning are paramount for our understanding of how the heart construes various stimuli, incorporates and orchestrates these into signaling cascades and alters gene expression to adapt to these stimuli. Modulation of interactions and crosstalk between cellular effectors, downstream targets in regard to gene expression as well as modification of the perception of stressors may therefore hold promise for future therapeutic approaches rather than aiming for the intermediates in signaling themselves—in reminiscence of the Greek philosopher Sophocles who postulated: “Do not kill the messenger!”

## Protein maintenance and turnover as critical setscrews of remodeling

### Growing tired (?)—get a shrink before burn-out!

As mentioned above cardiac plasticity covers a wide and dynamic range, which allows for distinct and impressive adaptations to altered hemodynamics and demands. Key determinants of cardiac homeostasis in this regard include mechanisms which govern the dynamics of protein synthesis and degradation, thereby creating an adequate equilibrium suitable for any given situation. Over the past decades, research has mainly focused on protein synthesis with its respective appendices, adaptations, modulations and control mechanisms in an effort to elucidate therapeutic potential of modifications of key mediators in these signaling pathways. In recent years, accumulating experimental evidence has led to a gradual shift in perspective (Hill and Olson, 2008; Portbury et al., 2011; Willis et al., 2014). While modes of protein synthesis and their modulation have remained as critical pillars in cardiac remodeling research, interest in mechanisms of degradation and atrophy has steadily grown (Hill and Olson, 2008; Portbury et al., 2012; Lavandero et al., 2013, 2015; Willis and Patterson, 2013; Willis et al., 2014). This is based on the notion that protein synthesis and degradation—while seemingly at opposing poles of cellular regulatory mechanisms—are in reality closely associated and hugely intertwined balancing mechanisms that guarantee adequate cellular responses and homeostasis (Willis and Patterson, 2013).

Therefore, the second part of our review will focus on this shift in perspective and the essential contributions of degradation and proteolysis to cardiac plasticity and homeostasis.

The cell is in dire need of tools which ensure adequate removal of misfolded or no longer-needed proteins as well as mechanisms that allow for well-controlled and regulated conversion and remodeling of its structural and functional units in response to intrinsic and extrinsic stimuli (Willis and Patterson, 2013). This deserves special emphasis in regard to the heart, more specifically cardiomyocytes, as these cells are terminally differentiated and characterized by a very limited regenerative potential (Willis and Patterson, 2013). Therefore, cardiomyocyte homeostasis, health and survival rely heavily on maintenance of protein quality control as well as critical balancing of synthesis, folding and turnover of proteins (Willis et al., 2009a). Cellular mechanisms to achieve this equilibrium include molecular chaperones and folding enzymes (nicely reviewed in Hartl et al., 2011; Christians et al., 2014 and therefore only briefly touched upon here) and coordinated systems for degradation and proteolysis, mainly autophagy and the ubiquitin-proteasome system (UPS) (Willis and Patterson, 2013; Willis et al., 2014; Lavandero et al., 2015)—elaborated on below. Only briefly mentioned here, but nevertheless very interestingly, SUMOylation has emerged as another important factor in regard to modification of protein turnover and homeostasis (Gupta et al., 2014).

Initially coined by Belgian biochemist Christian de Duve, the term “autophagy” describes the basic cellular approach to degrade and recycle dysfunctional or unnecessary components by lysosomal digestion (Blobel, 2013; Ohsumi, 2014). In this process, target structures are segregated into autophagosomes, double-membrane vesicles, which are then amalgamated to lysosomes. Consequently, lysosomal hydrolases mediate the breakdown and recycling of target structures resulting in cellular clearance (Lavandero et al., 2013, 2015; Ohsumi, 2014).

The Ubiquitin-Proteasome System is the second main effector of cellular degradation (Willis and Patterson, 2013; Willis et al., 2014; Drews and Taegtmeyer, 2014). While autophagy generally tackles (larger) aggregates, fractions or whole organelles, the UPS functions as a highly specific and meticulously tuned machinery by targeting single molecules that need to be degraded (Willis et al., 2014). Upon canonical labeling of lysine residues with ubiquitin chains, dispensable proteins are recognized and subsequently degraded by the 26S proteasome, a ubiquitous protease, marking the catalytic destination of the UPS pathway. Substrate-specificity of UPS-mediated degradation is achieved by a myriad of different ubiquitin ligases, which exhibit idiosyncratic binding sites tailored to their individual targets. Interestingly, downregulation of CSN (COP9 signalosome) confers a general reduction of F-box proteins, an important group of ubiquitin ligases, resulting in cardiac hypertrophy and severe heart failure (Su et al., 2011). This observation nicely depicts the homeostatic role of ubiquitin ligases in cardiac remodeling.

Adding another layer of complexity, various deubiquitinating enzymes may modify or even counteract the action of E3 ubiquitin ligases (Bosch-Comas et al., 2006; Bernardi et al., 2013).

There is significant crosstalk, cooperation and interdependency between the two basic mechanisms of cardiac proteolysis. In this regard, overload of and impairments in the UPS lead to compensatory upregulation of autophagy as an emergency solution or bypass to cope with the overflow. Yet, if both major systems are overwhelmed and failing (hypertrophic) remodeling accelerates and cardiac function declines (Tannous et al., 2008a,b; Su et al., 2011; Willis and Patterson, 2013).

Substrate-specific recognition by E3-ligases and subsequent degradation not only allow for the disposal of misfolded, damaged or no longer necessary proteins but offer a finely tuned and orchestrated means to direct cellular signaling. The cardiac sarcomere deserves special emphasis in this regard, as it serves as a pivotal node of signaling integrating stretch-responsive messages originating from the cell membrane, calcium-derived mechanisms of excitation-contraction (EC) coupling as well as multiple other cellular pathways which modulate the heart's quintessential function: force-generation for contraction of blood to supply the body with oxygen (Frank and Frey, 2011; Knoll et al., 2011; Portbury et al., 2011; van Berlo et al., 2013).

Illustrating this notion, most of the cardiac ubiquitin ligases identified to date target key sarcomeric proteins or molecules entangled with its associated network of signal effectors. For instance, the muscle-specific E3-ubiquitin ligase atrogin has been shown to ubiquitinate and thus to facilitate degradation of calcineurin (Li et al., 2004) which—as detailed above—represents one of the crucial enzymes in mediation of cardiac remodeling. Moreover, atrogin may exert its signaling effects in regard to hypertrophic remodeling by inducing the FoxO-transcription factor through non-canonical K63-ubiquitination (Li et al., 2007). FoxO in turn may induce atrogin (Sandri et al., 2004), while evidence suggests that at least in skeletal muscle both of the aforementioned (FoxO and atrogin) are additionally regulated by HDACs (Potthoff et al., 2007; Moresi et al., 2010). This may serve as a beautiful illustration of the complex crosstalk and interdependency of the UPS and key signaling elements of the cell.

Furthermore, the cardiac muscle-RING finger proteins (MuRFs) target parts of the contractile apparatus such as troponin I (MuRF-1) and myosin heavy chain (MuRF-1 and −3). In congruence, MuRFs (especially MuRF-1) have been shown as essential modulators of the hypertrophic remodeling process (Willis et al., 2007, 2009a,b, 2014; Fielitz et al., 2007a,b; Portbury et al., 2011; Willis and Patterson, 2013). Of note, the classic pathological vs. physiological dichotomy observed in research on other key players of myocardial remodeling is not commonly attributed in UPS research, where seemingly contradictive or at least multi-layered data have created the picture of gradual continua of context- and stimulus-specific rather than ligase-specific reactions and pathways. This complex nature of UPS-mediated reactions to cardiac stress is marvelously illustrated by contrasting works from Willis and colleagues: on the one hand transgenic overexpression of MuRF-1 mediates cardioprotective effects in models of ischemia, on the other hand MuRF-1 transgenic mice demonstrate progressive decline in cardiac function as well as increased susceptibility to TAC-induced heart failure (Willis et al., 2007, 2009b). Additionally, studies by Fielitz et al. show a loss of MuRF-3 to be detrimental as this deficiency renders the heart more prone to rupturing after myocardial infarction (Fielitz et al., 2007b). Furthermore, these mice deficient of MuRF-1 and MuRF-3 show increased susceptibility for adverse hypertrophic remodeling along with findings of storage and aggregate myopathies (Fielitz et al., 2007a). The high redundancy of E3 ligases in regard to their respective targets as well as the critical involvement of the UPS in terms of metabolism add even more layers of complexity (Adams et al., 2008; Witt et al., 2008; Willis et al., 2014). Intriguingly, further downstream proteasome modification itself can also contribute to or even sway the nature of cardiac remodeling processes (Drews et al., 2010).

A second example of the absence of a black-and-white dualism can be derived from data on atrogin in settings of myocardial remodeling:

While the observation that atrogin-transgenic mice are protected from TAC-induced pathologic cardiac hypertrophy is promising, it is remarkable that the very same mice are still prone to TAC-mediated deterioration of cardiac function on the other hand (Li et al., 2004; Portbury et al., 2012). Furthermore, atrogin-knockout mice exhibit both protection from pathologic hypertrophic remodeling and resistance to pressure-overload induced cardiac dysfunction (Usui et al., 2011).

These observations offer a conundrum that clearly opposes the classic partition into solely “good” or “bad” players that has been used in regard to other mediators of cardiac remodeling in the past.

Myofibrillar myopathies (MFM)/desmin-related myopathies (DRM), generally clustered as a subgroup of the heterogeneous group of dilated cardiomyopathies (DCM), also highlight the critical implications of the UPS for remodeling research. They prove beyond doubt that loss of degradation capacities—which is generally due to mutations in sarcomeric proteins, that render these mutated proteins less stable in the constant wear and tear, or even cause them to be misfolded from the beginning—is indeed causative and crucial for the development of hypertrophic remodeling and deterioration of cardiac function in its wake (Kley et al., 2013; Willis et al., 2014; Willis and Patterson, 2013; Schlossarek et al., 2014a). Desminopathy and filaminopathy are the best described representatives of these MFMs/DCMs. For the group of desminopathies, mutations in either desmin or its molecular chaperone (CryAB) have been shown to be associated with impaired autosomal degradation of the respective aggregates. Identification of mechanisms to enhance UPS- and autophagy-mediated elimination of these devastating aggregates of misfolded proteins appears paramount to counter adverse remodeling and consequent heart failure (Willis and Patterson, 2013; Willis et al., 2014; Schlossarek et al., 2014a). An exploratory study by Cohen and colleagues who analyzed filament composition and breakdown in atrophying muscle upon fasting suggests involvement of Trim32 in desmin ubiquitylation and degradation (Cohen et al., 2012). It nicely illustrates the need for further comprehensive *in vivo* studies to delineate the roles of Trim32 and other ubiquitin ligases as suitable catalysts for degradation of WT and mutant desmin and its appendices as the prototypical proteins of DCM/DRM.

Filaminopathies deriving from mutated filamin entities may also be promising objectives in UPS research, as two cardiac ubiquitin ligases, MuRF-3 and fbxl22, have so far been identified to target filamin C for proteasomal degradation and upregulation of these E3 ligases may therefore offer great therapeutic potential in this disease (Fielitz et al., 2007b; Spaich et al., 2012; Kley et al., 2013; Willis et al., 2014).

Much the same applies for other types of cardiomyopathies that derive from mutated sarcomeric proteins. For instance, numerous studies demonstrate that mutations in cardiac myosin binding protein C (cMyBPC) lead to hypertrophic cardiomyopathy (HCM) associated with impairment of the UPS, establishing cMyBPC as the paradigm of HCM-studies (Schlossarek et al., 2014a,b). Conversely and illustrative of the complexity of achieving homeostasis in regard to protein turnover and maintenance, proteasomal inhibition appears to ameliorate the decline in cardiac function in a hypertrophic cardiomyopathy model of mutated cMyBPC (Schlossarek et al., 2014b). While MuRF-1 has been shown to reduce cMyBPC levels, this occurs independent of its ubiquitin ligase activity and is likely due to indirect effects (Mearini et al., 2010). Furthermore, promising observations from *in vitro* studies demonstrate atrogin to be involved in degradation of mutated cMyBPC. On the downside, atrogin fails to target WT cMyBPC—potentially due to divergent localization of mutant cMyBPC (Mearini et al., 2010). These findings underscore that the underlying mechanisms and their therapeutic potential remain largely elusive and should be targeted by future research, along with intensive efforts to identify further E3 ligases involved in cMyBPC degradation.

The vast field of cardiomyopathies has seen significant advances in identification of causative gene mutations along with respective phenotyping. However, in many cases the implications of these mutations in regard to the UPS have not been understood yet and are far from utilization of the therapeutic potential which a distinct mapping of culprits and mutated target genes to respective ubiquitin ligases would harbor (Willis et al., 2014; Drews and Taegtmeyer, 2014; Schlossarek et al., 2014a). For instance, alpha-actinin, a key sarcomeric protein in terms of structure and signaling, has repeatedly been implicated in cardiac remodeling and disease development (DCM) but so far only *in vitro* data exists on its mode of degradation (Spaich et al., 2012). While the observation that the cardiac E3 ligase fbxl22 facilitates the degradation of alpha-actinin is auspicious, it plainly exemplifies and advocates, yet again, the need for future remodeling research to shift in perspective from solely analyzing the side of protein synthesis to minutely identifying and characterizing how each and every cardiac protein is regulated in terms of its homeostatic equilibrium and turnover.

Taken together this exemplary but to no end comprehensive presentation of autophagy- and UPS-related cardiac disease entities and mechanisms is supposed to reflect the multifaceted involvement and key roles of the degradation machinery in cardiac remodeling and disease development.

## Conclusion and future perspective

Finally, to integrate both parts of our review we will provide two examples that nicely illustrate and thus emphasize the intertwining character of cardiac remodeling with its complexity and diverse crosstalk mechanisms:

The first part of our review detailed evolving controversies on major cardiac signaling pathways and their key protagonists—namely calcineurin, PKA and CaMKII—while the second part focused on the idea that not only protein synthesis but also protein turnover and degradation are paramount for cellular homeostasis. Despite huge efforts in both fields of research to enhance our understanding how cardiac remodeling processes are governed, it seems surprising that at least for two of the formerly mentioned pivotal mediators of cardiac remodeling, PKA and CaMKII, knowledge in regard to the regulation of their turnover and degradation remains very scarce. To the best of our minds, no ubiquitin ligase has yet been identified and proven as a critical regulator of CaMKII. In terms of PKA ubiquitylation and degradation, only the ubiquitin ligase Praja2 has so far been shown to associate with and ubiquitinate the regulatory subunit of PKA in specific compartments (Lignitto et al., 2011). Furthermore, with atrogin and MuRF-1 only two ubiquitin ligases have been validated to perform E3 ligase activity upon calcineurin thereby targeting it for proteasomal degradation (Li et al., 2004; Maejima et al., 2014; Schlossarek et al., 2014a).

In light of the multiplicity and redundancy seen for other key cellular proteins (p53 is targeted by at least 10 different ubiquitin ligases, proteasomal degradation of c-Jun has been shown to be conferred by at least 4 different E3 ligases), it appears very likely that a number of ubiquitin ligases targeting PKA, CaMKII and CnA are still unidentified and may be very important for a comprehensive picture of the mechanisms that control cardiac remodeling (Portbury et al., 2012; Willis et al., 2014; Maejima et al., 2014).

The second interesting notion highlighting the integrative and highly complex mechanisms that govern cardiac plasticity and remodeling concerns the observation that exercise, therefore physiologic hypertrophic stimuli, may alleviate myofibrillar myopathies and proteotoxicity in the wake of UPS impairments and dysfunction (pathological cardiac remodeling) or lead to improvement of UPS function itself (Maloyan et al., 2007; Willis and Patterson, 2013; de Andrade et al., 2015). This intriguing interdependency demands for further clarification of the underlying crosstalk to enhance our understanding how induction of certain mediators by exercise may provide tools for renewal of cellular homeostasis in failing myocardium.

**Figure 2:**
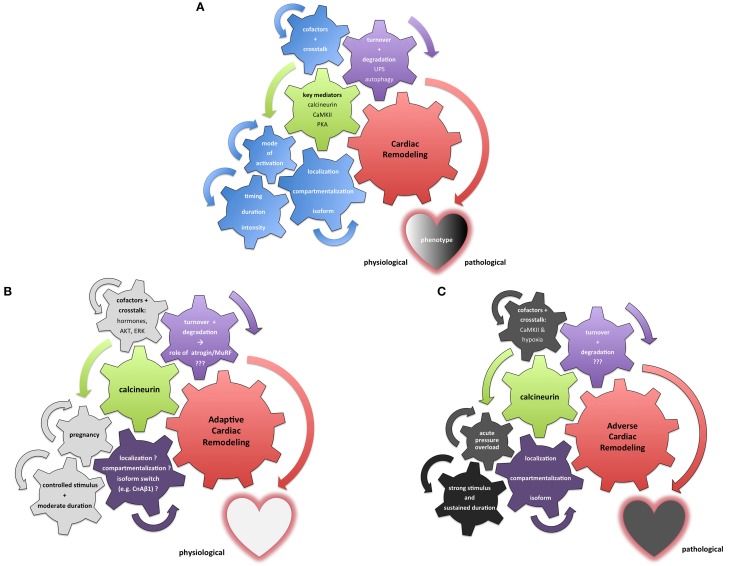
**Integrative illustration of the complexity and context-sensitivity of cardiac remodeling**. **(A)** Neutral illustration how various setscrews determine the result of cardiac remodeling processes. Multiple pivotal molecules such as calcineurin, PKA and CaMKII have been identified as key mediators of myocardial signaling (green) in the remodeling process (red). A dualistic perspective of “*bad*” and “*good*” mediators fails to integrate the myriad of context-dependent stimuli and crosstalk mechanisms (depicted as the blue setscrews in this illustration). Furthermore, protein maintenance and degradation (purple) contribute to the dignity and nature of cardiac remodeling processes. **(B)** Exemplary illustration of calcineurin's role in adaptive cardiac remodeling processes that result in a beneficial phenotype. While a number of stimuli and setscrews remain only incompletely understood or even elusive so far (purple), multiple relevant extrinsic and intrinsic factors potentially modulate calcineurin signaling toward a more beneficial response (depicted in light gray). Pregnancy-associated cardiac remodeling has been shown to be beneficial in nature; hormonal and temporal factors depicted here appear to contribute significantly to this adaptive dignity of calcineurin signaling. **(C)** Contrasting illustration of calcineurin's role in a maladaptive setting promoting cardiac remodeling processes that result in an adverse phenotype. While multiple factors—especially in regard to protein turnover—remain only incompletely understood or even elusive so far (purple), numerous extrinsic and intrinsic stimuli and stressors drive calcineurin signaling toward pathological effects with an adverse cardiac phenotype (depicted in dark gray). The pressure overload phenotype has been intensively studied; evidence suggests that calcineurin's signaling is swayed toward a maladaptive course not only by sudden onset, pronounced duration and intensity of the remodeling stimulus but also by cofactors such as activated CaMKII.

In any case, this observation underscores the concept that seemingly distant stimuli and protagonists of myocardial signaling (exercise on the one hand, the UPS on the other hand) intricately affect each other and prove the cardiac remodeling processes to be comprehensive and minutely tuned programs that integrate all cellular pathways into a highly plastic and flexible machinery (Figure 2).

In summary, this review provides some thought-provoking impulses on emerging or ongoing controversies and shifts in perspective surrounding myocardial remodeling rather than trying to comprehensively cover the myriad of elaborate mechanisms of cardiac signaling.

Therefore the two key messages are:

1. Protagonists of cardiac remodeling demonstrate multifaceted crosstalk and significant interdependency in a highly context-sensitive nature. Therefore it is our opinion that they cannot be easily classified in terms of a black and white dualism but appear to roam a gradual continuum of “fifty (or more) shades of gray.”
2. While protein synthesis and its regulation remain paramount for our understanding of cardiac adaptations, a shift in perspective has occurred in recent years as the mechanisms of maintenance and turnover of proteins have increasingly been recognized as essential aspects of sustained homeostasis—or to put it in other words: “Growing tired (?)—get a shrink before burn-out!”

### Conflict of interest statement

The authors declare that the research was conducted in the absence of any commercial or financial relationships that could be construed as a potential conflict of interest.

## Abbreviations

AKT: Protein kinase B (PKB)
ANF: atrial natriuretic factor
βAR: beta-adrenergic receptor
BNP: brain natriuretic peptide
CaMKII: Calcium/Calmodulin-dependent Kinase II
CnA: Calcineurin A
CnB: Calcineurin B
Cn: Calcineurin
cAMP: Cyclic adenosine monophosphate
DCM: Dilated Cardiomyopathy
DRM: Desmin-Related Cardiomyopathy
ERK: extracellular-signal-regulated kinases
Fbxl22: F-box and leucine-rich repeat protein 22
HCM: Hypertrophic Cardiomyopathy
HDAC: Histone Deacytelase
MEF-2: myocyte enhancer factor-2
MFM: Myofribrillar Myopathy
MuRF: Muscle Ring Finger Protein
NFAT: nuclear factor of activated T cells
PKA: Protein Kinase A
PKC: Protein Kinase C
RCAN: regulator of calcineurin
ROS: Reactive Oxygen Species
UPS: Ubiquitin Proteasome System.
